# Dissociative experiences in individuals with subclinical psychosis and a history of developmental trauma: a qualitative study

**DOI:** 10.1080/20008066.2025.2472473

**Published:** 2025-03-11

**Authors:** Eirini Aikaterini Melegkovits, Ava Mason, Jordan Reid, Hind Akooly, Paul Jung, Michael Bloomfield

**Affiliations:** Division of Psychiatry, University College London, London, UK

**Keywords:** Dissociation, developmental trauma, subclinical psychosis, psychotic experiences, qualitative study, thematic analysis, Disociación, trauma del desarrollo, despersonalización, psicosis, psicosis subclínica, maltrato, experiencias psicóticas, cualitativo, análisis temático

## Abstract

**Background:** Among individuals with psychotic experiences, those with a history of developmental trauma face greater symptom severity and worse clinical outcomes compared to those without. Dissociation constitutes a prominent, characteristic of this group’s clinical presentation, whose nuances and associated characteristics remain however understudied in psychosis research. We aimed to address this gap by conducting a qualitative study to investigate the phenomenology, context, and impact of dissociative experiences in individuals with subclinical psychosis and a history of developmental trauma.

**Methods:** 25 UK-based participants with a history of developmental trauma and meeting criteria for subclinical psychosis, based on the CAPE-15, were recruited via social media. Participants attended semi-structured interviews online, which were transcribed verbatim and analysed with thematic analysis by two researchers.

**Results:** Thematic analysis yielded the following themes: (1) Phenomenology of Dissociation; (2) Context of Dissociation; (3) Impact of Dissociation; (4) Dissociation and Psychotic-like Phenomena. Participants described experiences of detachment and compartmentalisation, which when experienced were confusing and often distressing. Dissociation was linked to affective experiences, their history of developmental trauma and psychotic-like experiences.

**Conclusion:** This study elucidates the complex and varied nature of dissociative experiences in individuals with subclinical psychosis with a history of developmental trauma. These findings highlight the need for further research to understand the manifestation of dissociation in this population and the links with distress and other aspects of psychopathology. Importantly, it is essential to use this understanding to inform the development of interventions and improve clinical recognition and management.

## HIGHLIGHTS

- Dissociative experiences, and especially depersonalisation, are a frequent and highly heterogeneous experience among individuals with subclinical psychotic symptoms and developmental trauma.
- Dissociation is identified as a way of coping at the time of developmental trauma, but also as a response to post-traumatic intrusions, intense and distressing emotions and hallucinations.
- Dissociative experiences elicit feelings of panic and helplessness, and are linked to the occurrence of psychotic-like experiences, such as hallucinations and mistrust of others.

## Abstract (es)

**Antecedentes:** Entre los individuos con experiencias psicóticas, aquellos con una historia de trauma del desarrollo se enfrentan a una mayor gravedad de los síntomas y peores resultados clínicos en comparación con los que no la tienen. La disociación constituye una característica prominente de la presentación clínica de este grupo, cuyos matices y características asociadas permanecen, sin embargo, poco estudiados en la investigación de la psicosis. Nuestro objetivo fue abordar esta brecha mediante la realización de un estudio cualitativo para investigar la fenomenología, el contexto y el impacto de las experiencias disociativas en individuos con psicosis subclínica y una historia de trauma del desarrollo.

**Métodos:** 25 participantes del Reino Unido con antecedentes de trauma del desarrollo y que cumplían los criterios de psicosis subclínica, basados en el CAPE-15, fueron reclutados a través de las redes sociales. Los participantes asistieron a entrevistas semiestructuradas en línea, que fueron transcritas literalmente y analizadas con análisis temático por dos investigadores.

**Resultados:** El análisis temático arrojó los siguientes temas: (1) Fenomenología de la disociación; (2) Contexto de la disociación; (3) Impacto de la disociación; (4) Disociación y fenómenos de tipo psicótico. Los participantes describieron experiencias de distanciamiento y compartimentación que, cuando se experimentaban, resultaban confusas y a menudo perturbadoras. La disociación se relacionó con experiencias afecs, su historia de trauma del desarrollo y experiencias de tipo psicótico.

**Conclusiones:** Este estudio elucida la naturaleza compleja y variada de las experiencias disociativas en individuos con psicosis subclínica con historia de trauma del desarrollo. Estos resultados ponen de relieve la necesidad de seguir investigando para comprender la manifestación de la disociación en esta población y los vínculos con el malestar psicológico y otros aspectos de la psicopatología. Es importante destacar que es esencial utilizar esta comprensión para fundamentar el desarrollo de intervenciones y mejorar el reconocimiento y el manejo clínicos.

## 1. Introduction

The link between developmental trauma (DT) and psychosis is well-documented, with individuals who have experienced childhood abuse or neglect facing increased risk of psychosis (Kelleher et al., 2013; Varese et al. (2012), more severe psychotic symptoms (Bailey et al., 2018; Onyeama et al., 2024), and worse prognostic and treatment outcomes (Schenkel et al., 2005; Thomas et al., 2019). Dissociation is a common feature of the clinical presentation of psychosis (Longden et al., 2020), and is elevated in patients who additionally have a history of DT (Rafiq et al., 2018).

Dissociation has been described as a complex alteration of conscious awareness. It is often characterised by a disconnection from aspects of one’s experience of the self or the environment, as seen in forms of dissociative detachment (Holmes et al., 2005). Detachment encompasses experiences of depersonalisation, a feeling of disconnection from bodily and emotional experience, and derealization, the perception of the world as unreal or strange. Dissociation may also involve a separation or ‘splitting’ of psychological functions and structures (van der Hart et al., 2006) such as ‘consciousness, memory, identity, emotion, perception, body representation, motor control, and behaviour’ (American Psychiatric Association, 2013, p. 291), noted in compartmentalisation (Holmes et al., 2005). Notably, dissociative phenomena range in intensity and severity, from common everyday events such as daydreaming to pathological and highly distressing incidents of out-of-body experiences, feelings of a loss of self, or dissociative identity disorder.

It is frequently documented that dissociation can emerge as a way of coping during or in the aftermath of trauma (van der Hart et al., 2006), offering a mental escape from an overwhelming, physically and psychologically painful experience (Foa & Hearst-Ikeda, 1996; Haugaard, 2004; Putnam, 1992). Individuals traumatised in childhood may exhibit greater disrupted attachment and lower mentalization (i.e. the unique ability to imagine the intentions that may underlie the behaviour and affective presentation of oneself and others) (Ensink et al., 2017; Fonagy et al., 2000; Liotti & Gumley, 2008; Wagner-Skacel et al., 2022), which have been associated with the higher levels of dissociation exhibited in this group (Vonderlin et al., 2018).

Although dissociative experiences are highly prevalent in psychosis (Alameda et al., 2020; Bloomfield et al., 2021; Longden et al., 2020; Pilton et al., 2015) they often remain unrecognised, or confused with psychotic features or other conditions. This lack of recognition becomes complicated due to different theoretical conceptualisations of dissociation, along with high symptom overlap (Renard et al., 2017). For example, manifestations of depersonalisation involving emotional numbing are similar to aspects of emotional withdrawal and blunted affect characterising negative symptoms of psychosis, and metaphorical descriptions of dissociation may be interpreted as unusual beliefs observed in psychosis.

This lack of recognition in clinical settings may underpin the significant knowledge gap regarding the treatment of dissociation in psychosis, which further complicates the design and delivery of clinical interventions. Dissociation has further been proposed as a putative mechanism underlying the relationship between trauma and positive symptoms in psychosis, particularly hallucinations (Alameda et al., 2020; Bloomfield et al., 2021; Varese et al., 2012). On the other hand, some authors have suggested that psychotic symptoms, especially among those with histories of trauma, may in fact represent dissociated expressions of compartmentalised material (Brewin et al., 2022; Moskowitz & Corstens, 2008) such as memories of traumatic events in the form of hallucinations. Upon reviewing the research literature, one noteworthy limitation is the lack of exploration of dissociation in the context of subclinical psychosis, often referred to as ultra-high risk for psychosis or at-risk mental states, despite clinical accounts of dissociation encountered frequently in the psychosis prodrome. Studying the developmental trajectory of experiences central to subclinical or emerging psychosis, and capturing the interaction of dissociation with DT, can enhance our understanding and clarify the clinical utility of screening for dissociation as a prevention measure for future psychotic episodes.

Research to date linking psychosis, trauma, and dissociation has predominantly relied on quantitative correlational research, which may fail to capture the lived aspects of these experiences, the contexts in which they arise, and the directionality of their complex links. Qualitative research, contrastingly, offers a chance to delve into individuals’ phenomenological experiences, creating opportunities to garner new insights that could be of great clinical utility in this unexplored phenomenon. To our knowledge, only one qualitative study has provided qualitative accounts of dissociation in psychosis (Černis et al., 2020), where participants characterised dissociating as unsettling and so challenging to put into words that it feels indescribable. However, the authors (Černis et al., 2020) did not screen or directly enquire for trauma history, and focused on individuals with clinical levels of psychosis.

To address this important gap in the literature, the present study aimed to understand the phenomenological experience of dissociation among participants with subclinical psychosis and a history of DT, including any associated psychopathological mechanisms, using qualitative interviews. Further, we aimed to identify links between dissociative experiences and DT history, but also affective and psychotic experiences to inform the future development of formulation and treatment.

## 2. Methods

### 2.1. Ethical considerations

This study forms part of the Investigating Mechanisms Underlying Psychosis Associated with Childhood Trauma study. Ethical approval was sought from the UCL Research Committee (Ref: 17495/001). The Declaration of Helsinki and Good Clinical Practice were stringently adhered to throughout the research.

### 2.2. Participants

Participants were recruited online as part of the community arm of the larger ‘IMPACT’ study (see Supplementary Material 1 for further information on recruitment) and were contacted to participate in this qualitative study via email. Inclusion criteria were >18 years old, a UK resident and fluent in English. All participants had a history of DT, which was determined the Childhood Trauma Questionnaire (CTQ) (Bernstein & Fink, 1998). In order to ensure specificity and minimise risk of over-reporting, we required a score of moderate and above on two items or severe and above on one item of the CTQ following the cut-off criteria by Bernstein and Fink (1998). All participants screened above threshold for Ultra-High Risk of Psychosis on the Community Assessment of Psychic Experiences (CAPE)-15 (Capra et al., 2013; Sun et al., 2020), using the validated cut-off of an average score of 1.47 across items of the frequency and distress subscales. Descending scores guided the selection process on both scales, whereby participants with the highest weighted CAPE-15 score and highest CTQ scores were invited first to ensure the presence of DT and subclinical psychotic symptoms in the sample.

### 2.3. Interview schedule

The semi-structured interviews consisted of two parts. First, participants responded to the short version of the Comprehensive Assessment of At-Risk Mental States (CAARMS) (Yung et al., 2005), a semi-structured interview assessing UHR status by measuring a range of sub-threshold psychotic-like symptoms. The CAARMS includes a section dedicated to exploring dissociative symptoms, namely depersonalisation, derealisation and dissociative amnesia*.* In addition, some CAARMS questions measuring unusual thought content, visual or somatic changes are known to elicit responses pertaining to dissociation, and these are described in more detail in Supplementary material 2. The second part consisted of a tailored version of the Intrusions Interview (Patel et al., 2007), a semi-structured interview exploring two recent trauma-related intrusive memories, images, thoughts or voices and their impact on daily functioning among people with psychosis (Supplementary Material 3). Follow-up questions on the phenomenology of the dissociative and psychotic experiences described, intrusive memories and images, and questions on the response to these experiences were used to elicit more detailed descriptions of the experiences provided as responses to both the CAARMS and intrusions interview.

### 2.4. Procedures

All participants were informed of the anonymous and confidential nature of the study upon invitation, and completed written informed consent, including for the purposes of audio recording. All online interviews were audio recorded via video conferencing platforms due to the COVID-19 restrictions in 2020. Interview duration lasted between one to three hours, and participants were reimbursed £10.75 per hour for participation. Interviews were transcribed manually verbatim by two researchers (AM, EM).

Subsequently, interviews were coded using NVivo 12 for Mac (Ltd, Q. I. P., 2012). Given the study’s explorative nature of dissociation in relation to DT and psychosis, and the absence of previous qualitative studies on trauma and dissociation among those with subclinical psychotic symptoms, we opted for Thematic Analysis (TA), and specifically a hybrid inductive deductive approach, to analyse the data (Braun & Clarke, 2006), allowing flexibility but rigour to explore this understudied phenomenon. A hybrid approach synthesises both the top-down deductive approach which are found on theory, and the bottom-up data-based approach (Swain, 2024), allowing for new and unanticipated themes to emerge, but also themes aligning with previous research. This approach has been used in previous qualitative studies on trauma and psychosis (Campodonico et al., 2022).

In line with the first step of TA, coders familiarised and immersed themselves with the data by first reading the manuscripts (Braun & Clarke, 2006). The first author (EM) coded all 25 transcripts, while a second coder (HA) analysed 10% of the transcripts, and codes were checked to ensure inter-rater reliability. As transcripts were read, codes were grouped into subthemes and themes through discussion between coders and modified with the addition of new transcripts. Moreover, regular meetings were held among coders to ensure the precision and consistency of the analysis and resolve any incongruities.

## 3. Results

Sample sociodemographic and clinical characteristics are described in Table 1. The final sample was 68.00% female (*n* = 17) between the ages of aged 21–40 (*M* = 26.84, SD = 5.05). The majority identified as White British. On average, our sample reported severe to extreme levels of emotional abuse, low to moderate levels of physical abuse, and moderate to severe levels of sexual abuse, physical and emotional neglect, based on the CTQ. All participants had experienced at least one form of abuse at moderate levels, and 88% had experienced one form of neglect. Most participants reported experiencing multiple traumatic experiences, with 88% of participants experiencing at least two traumas.

**Table 1.** Sample sociodemographic and clinical characteristics.

Sociodemographic and Clinical Characteristic	*n* (%)
Age [M(SD)]	26.84 (5.05)
Female	17 (68.00%)
Ethnicity	
White British	15 (60.00%)
White other	6 (24.00%)
Asian	2 (8.0%)
Berber-Moroccan	1 (4.00%)
Educational attainment	
No qualifications	1 (4.35)
GCSE's or equivalent	3 (12.00%)
Other higher education	2 (8.00%)
Bachelor’s degree and above	19 (76.00%)
Family history of psychiatric disorder	15 (60.00%)
Previous use of psychiatric medication	17 (68.00%)
Number of Traumatic events^a^	*n* (%)
1	3 (12.00%)
2	5 (20.00%)
3	3 (12.00%)
4	9 (36.00%)
5	5 (20.00%)
Childhood Trauma Questionnaire	M (SD), [min-max]
Physical abuse	10.96 (5.56), [5–22]
Sexual abuse	11.95 (7.09), [5–25]
Emotional abuse	19.56 (4.07), [11–25]
Physical neglect	11.56 (4.23), [5–23]
Emotional neglect	17.39 (4.39), [7–25]
Total score	71.43 (19.00)
CAPE^b^-15 Frequency Total Score [M(SD)]	30.36 (7.41)
CAPE-15 Distress Total Score [M(SD)]	37.56 (11.49)
Complex PTSD ^c^ [M(SD)]	32.12 (12.09)

^a^ Traumatic Events rated as moderate on the Childhood Trauma Questionnaire.

^b^ CAPE-15 = Community Assessment of Psychic Experiences – Positive 15-items Scale.

^c^ Complex PTSD measured via the International Trauma Questionnaire.

The average CAPE-15 weighted score was *M* = 2.02 (SD = 0.48) for frequency of positive psychotic symptoms and *M* = 2.5 (SD = 0.75) for distress from psychotic symptoms. This indicates psychotic symptoms were experienced often, and were on average ‘often’ to ‘nearly always’ distressing. Two participants met threshold for post-traumatic stress disorder and eleven individuals met criteria for complex post-traumatic stress disorder, based on the International Trauma Questionnaire (Cloitre et al., 2018). A chi square test of independence showed no significant association between rates of PTSD/CPTSD and endorsing dissociative experiences during the interviews.

Depersonalisation was described by 14/25 participants, followed by derealisation phenomena, which were described by 9/25 participants, of which 7 also experienced depersonalisation. Dissociative amnesia was noted by 4/25 participants, all of whom also described depersonalisation phenomena, were female and met criteria for CPTSD.

The thematic analysis yielded four themes that are described below (see Tables 2–5).

**Table 2.** Theme 1: Phenomenological characteristics of dissociation.

1a. Awareness, control and ownership of bodily experience	‘It is a physical sense of detachment … it’s almost like a retreat basically … I’m watching out through my eyes and hearing what I’m saying but I'm not properly physically feeling stuff’ (P24) ‘Sometimes when I'm doing things with my hands it feels like these are not my hands, it doesn't feel like I'm doing them’ (P19) ‘My body doesn’t feel like it’s mine … it feels like I'm piloting a meat sack’ (P6) ‘My eyes, my head, my goddamn arms. I feel like I've got to take everything out and then just suddenly re-sew it back up or something, I don't know. It's weird.’ (P2) ‘I feel like most of my limbs have a mind of their own. It’s almost like a subset of me rather than a separate part of me’ (P23) ‘Sometimes I can't make myself work my left hand like … . there's nothing physically wrong with it, but it just feels like I don't have control of the hand.’ (P12)
1b.Depersonalisation: ‘Looking at myself from the outside’	‘Just sat in bed and I was looking down on myself or … I was walking down this main road and I could just see myself from behind, as If I was walking behind me’ (P12) ‘I’m watching myself from behind, like in a film’ (P2) ‘It’s like I'm not here really, dissociated from my body and I'm looking at myself from the outside, but that's not me..’. (P19) ‘It’s not necessarily outside, but it just doesn't feel like I'm inside’ (P25) ‘I felt like playing a third person video game so but with yourself, so you're over your own shoulder and you're watching yourself do these things … .’ (P24) ‘I think that's the one that describes it best, I feel like a camera, you know, and not a person’ (P3)
1c. Detachment linked to emotional numbness	‘And just when I get to that sort of stage, when I’m shutting down mentally in the sense of I don't feel anything anymore’ (P12) ‘I feel like I feel numb when in normal people they would feel extremes of emotions’ (P19)
1d. Derealization phenomenology	‘I think it's when I come out … . If I've been disassociating and I stopped disassociating everything seems really big … Or when this happened everything was a bit weird ‘cause your kitchen has been missing’ (P16) ‘I find those occasions just puzzling to myself because I'm not used to looking at something and not really recognizing.’ (P22) ‘ … Places that I've been in before, or settings, you know, sometimes feel … not, not very familiar. I don't know how to say that.’ (P4) ‘It feels strange. I think it is a form of dissociation, it’s as if I'm in a game world, like everything's glitching. it doesn't feel complete or real.’ (P2)
1e. Compartmentalisation phenomenology	‘When my mind goes blank, and I disappear I then can’t remember what I was saying’ ‘Yes, yeah. I sometimes realise that I've completely blanked out. And I don't even know what I was thinking about, let alone what someone might have been saying to me.’ (P4) ‘There have been like times when you know sometimes when you're going somewhere on and you're thinking about something and then suddenly you forgot how you got there.’ (P22)

**Table 3.** Theme 2: The context of dissociation.

2a. Dissociation as a response to trauma in childhood	‘[After describing that surroundings feel unfamiliar] ‘Yeah, I think probably like the way that I coped [with emotional and physical trauma in childhood] was to just like switch off’ (P15)
2b. Dissociation following trauma reminders	‘When someone raises their voice … I am immediately out. It’s just like watching yourself through a TV screen.’ (P12) ‘When I have these mental images of what happened to me as a kid. I always feel I'm somehow removed from my physical body. It's really odd to come try and explain it.’ (P13) ‘I have moments where I remember stuff from my dad, my surroundings sort of shut off’ (P2)
2c. Dissociation in response to low mood and distressing emotions	‘There’s been quite a few times when I've been feeling quite low … I sometimes feel like I’m observing myself’ (P13) ‘A handful of times that would likely be due to extreme stress I guess. I got really angry and shocked … And then it felt I was watching over my shoulder’ (P24) ‘It usually happens if I'm surrounded by loads of people … It's very overwhelming for me. At some point, they sort of become background. And I'm a camera’ (P3)

**Table 4.** Theme 3: Impact of dissociative experiences.

3. Impact of Dissociative Experiences	‘Yeah, it (depersonalization) makes me feel like there's something wrong with me … like I'm not behaving normally like am I abnormal? It makes me a lot more like scared of not feeling anything’ (P19) ‘I just don't feel my body … a floating thing. And that's the worst really. You don't feel anything and you feel everything at the same time. It's very threatening … It triggers a panic attack’ (P3) ‘When I have trouble making my limbs do what I want them to it makes me feel panic’ (P18) It makes me feel frustrated … is this not going to stop … like this is this going to be around forever? This is just how it will be? (P15)

**Table 5.** Theme 4: Dissociation and psychotic-like experiences.

4a. Dissociation contributing to paranoia	‘When I’m in that switch off mode and my surroundings feel strange … It makes me become anxious and more mistrustful of others’ (P15) ’I felt very dissociated and not aware of myself … I thought that others might not be aware of me, questioning how others perceive me, if others perceive me’ (P21)
4b. Dissociation associated with perceptual anomalies and hallucinations	‘Sometimes things will be brighter in colour or there's more space. It relates to whether I’ve dissociated’ (P16) ‘It might be a reaction to what happened … [When remembering the trauma] my surroundings sort of shut off … . I have a sense of people holding my shoulders and stuff, or holding my mouth, and distorting my face and things’ (P2)
4c. Dissociation as a response to hallucinations	‘When I struggle with like voices and things, I don't know, that kind of helps in a sense.’ (P2) ‘[When I hear them] I get into myself so much and think why do I even bother? And I get to that stage of shutting down mentally, I don't feel anything anymore’ (P12)

### 3.1. Theme 1: Phenomenology of dissociation

This theme describes the phenomenological aspects of dissociative experiences identified by participants.

#### 1a. Depersonalisation: awareness, ownership and control of bodily experience

In this subtheme (see Table 2), eight participants described a range of changes in their embodied experience and their sense of body ownership. Three participants spoke about having less awareness and experience of their physical body. Six participants described a sense that their body or body parts did not belong to them, or a sense of body parts being ‘lifeless’. This included noticing this disconnection while doing activities or looking in the mirror and being confused about who they are seeing. Three participants further described a sense of not being able to control their body or body parts.

#### 1b. Depersonalisation: ‘looking at myself from the outside’

In this subtheme, nine participants described a sense of looking at themselves from ‘outside’ or from above, or not feeling that they are inside their body. Several participants used the analogy of a ‘film’, a ‘video game’ or ‘TV screen’.

#### 1c. Detachment linked to emotional numbness

Five participants also described a sense of emotional detachment, existing on its own or accompanying physical detachment. This emotional numbness was also described as an unexpected state to events that would ‘usually’ or ‘normally’ provoke an intense emotional reaction in other people.

#### 1d. Derealization phenomenology

Five participants provided descriptions of not recognising their surroundings or experiencing a sense of unfamiliarity. Some participants commented on elements of their surroundings appearing different to the point that they might be unrecognisable. Notably, of these two participants connected this feeling to experiences of depersonalisation, such as ‘being out’ or ‘switching off’.

#### 1e. Compartmentalisation phenomenology

Four participants described finding themselves in a place without remembering how they got there, or not remembering what they were saying or doing following what they recognised as dissociation. When this experience was explored further by the interviewer, all participants related it to depersonalisation phenomena, such as a feeling of ‘blankness’ or ‘disappearing’.

### 3.2. Theme 2: Context of dissociation

Participants (see Table 3) connected their experiences of dissociation, in particular depersonalisation and ‘shutting down’, to their experience of DT or having experienced peri-traumatic dissociation in childhood. Six participants also described dissociation occurring as a response to reminders of traumatic events and post-traumatic intrusions, such as memories of childhood trauma. Finally, participants spoke about experiencing dissociation as a state when low, distressed, worried and overwhelmed.

### 3.3. Theme 3: Impact of dissociative experiences

Seven participants (see Table 4) described dissociation as a highly distressing and threatening experience, which was phrased as being incomprehensible and confusing by six participants. Responses to these profoundly overwhelming sensory and emotional experiences involved panic and worry among seven participants. Three participants described metacognitive thoughts reflecting fear about ‘abnormality’ or how permanent and generalisable to other situations this experience might be.

### 3.4. Theme 4: Dissociation and psychotic-like phenomena

Five participants (see Table 5) described that acute dissociation and disconnection from their experiences increases their awareness of themselves and others, contributing to feelings of mistrust and paranoia. Dissociation was also linked to perceptual abnormalities among six participants, such as changes in shape, brightness and colour of objects, but also to tactile anomalous experiences akin to hallucinations. Finally, two participants described dissociation as a response to the distress induced by hallucinations.

## 4. Discussion

In the present study, we aimed to qualitatively explore the nature of dissociative experiences among individuals who experience subclinical psychosis and have a DT history. Using semi-structured qualitative interviews, we identified five themes: ‘Phenomenology of dissociation’; ‘The context of dissociation’; ‘The impact of dissociative experiences’ and ‘Dissociation and psychotic-like phenomena’. Overall, our sample described a range of detachment and compartmentalisation phenomena. Participants expressed strong emotional reactions to these occurrences, such as panic and hopelessness, and attempted to explain what often appeared inexplicable, tracing their present experience back to instances of abuse that profoundly changed their perception of themselves and the world. In this section we discuss findings in the context of the broader literature and arising clinical implications.

The first theme encompasses the phenomenology of dissociation. Of note, the majority of our sample experienced some form of dissociative experience. Although these experiences pertained mainly to depersonalisation phenomena, their manifestation was heterogeneous, capturing a multitude of different feelings of disembodiment, emotional numbing, and fewer instances of derealization and compartmentalisation. Although all participants who experienced dissociative amnesia met criteria for CPTSD based on the International Trauma Questionnaire, they did not directly relate their amnesic symptoms to any post-traumatic experiences. However, similar to a qualitative study by Černis et al., (2020), such incidents of compartmentalisation, i.e. disruptions in one's memory and sense of identity, were predominantly described along experiences of detachment. This distinction is relevant, as amnesia associated with detachment may indicate memory encoding deficits during a detached state rather than retrieval problems (Allen et al., 1999 as cited in Holmes et al., 2005).

Disembodiment was frequent and characterised by changes in awareness, body ownership and lack of control of body parts, and ranged in subtlety, from a sense of not occupying the body to several participants noting out-of-body experiences, aligned with past qualitative findings among participants with psychosis (Černis et al., 2020). Emotional numbing was also frequently described, similar to previous accounts of de-affectualization symptoms in depersonalisation (Medford, 2012). Other aspects of recounted depersonalisation experiences included feeling ‘cut off from the world’ or one’s body. These striking descriptions of disembodiment and derealisation were contextualised through the lens of trauma by many participants, reflecting a mind and body which became disconnected from each other, continuing to bear the scar of experiencing a caregiver as hostile, harmful and denigrating.

The language used among participants is akin to previous descriptions of dissociation (Černis et al., 2020; Sierra & David, 2011), alluding to a separation from reality and experience or a dream-like state. As in previous studies (Ciaunica et al., 2022; Černis et al., 2020; Sierra et al., 2005), participants found it hard to describe their subjective experiences, reported confusion and relied extensively on terms such as ‘as if … ’ and ‘like’ to find ways to communicate their subjective experience.

Participants also described the world appearing as ‘lifeless’, or people in it appearing as less real. Participants used visual metaphors of ‘cameras’, ‘videogames’, the presence of ‘shadows’ and a ‘haze’, reflecting a disconnection from the world, one that is ‘glitching’, and revealing the intensity of how incomprehensible the world feels after the devastating experience of abuse. This difficulty to convey their experience and use of metaphors are also highly clinically relevant, as they may contribute to both the under recognition of dissociative experiences in patients with psychosis and to the misinterpretation of metaphorical descriptions of dissociation for psychotic symptoms.

Consistent with earlier quantitative studies (Rafiq et al., 2018), participants connected their experience of dissociation to their trauma. Two distinct pathways emerged in these accounts: first, dissociation as an in the moment response to trauma and to disrupted caregiver attachment, and second, dissociation arising from the post-traumatic sequelae of DT. Participants described ‘shutting down’ as a primary coping mechanism in childhood. When children are exposed to trauma, dissociation can serve as a vital escape from danger and threats to their physical integrity (Fonagy et al., 2000), but also from the shock and devastation of abuse on the body, and accompanying confusion and shame. Furthermore, young children inherently rely on caregivers to learn about the world and employ epistemic vigilance to differentiate between reliable and malevolent sources of information (Fonagy et al., 2019). When this epistemic trust is fractured, the attachment relationship fails to offer comfort and support (Fonagy & Allison, 2014), and children may enter dissociative states to avoid contemplating the intentions and mental states of a threatening caregiver (Ensink et al., 2017; Huang et al., 2020).

Some participants described dissociative states elicited during situations that connected to traumatic events in childhood, revealing the impact of trauma on dissociation through memory processes. For example, some participants described dissociation triggered by sensory reminders of DT, such as someone raising their voice in a manner reminiscent of an abuser, or upon recollections such as confronting trauma-related images. These findings reflect dissociative manifestations akin to those in PTSD. The dual representation theory (Brewin, 2001; Brewin et al., 2010) posits that peritraumatic factors may interfere with the consolidation of contextual memory, resulting in trauma memories emerging as flashbacks. As identified by participants, peritraumatic dissociation may be one such factor, however, previous research points to subsequent persistent dissociation and suppression of processing traumatised memories as factors substantially aggravating post-traumatic stress symptoms (Briere et al., 2005).

Beyond the direct links between dissociation and trauma in childhood or adultdhood, participants also described dissociation in response to negative affective experiences (Foa & Hearst-Ikeda, 1996). Echoing qualitative findings by Černis et al. (2020), some participants described dissociation following ‘intense’ or ‘extreme’ emotions perceived as threatening or as exceeding their ability to cope. Previous studies propose that mechanisms such as affect intolerance (Černis et al., 2022a; 2022b), alexithymia (Evren et al., 2012; Černis et al., 2022a; 2022b), and diminished mentalizing capacity (Ensink et al., 2017; Wagner-Skacel et al., 2022) contribute to this phenomenon. As described above, adult survivors of DT may have frequently and unconsciously resorted to dissociation to avoid the internal and bodily sensations of physical and emotional pain, fear, shame, and guilt (Herman, 1998). Over time, reliance on dissociation may become a learned automatically generated (and for some the only) coping response in the face of threat or distressing emotions. Adding to this, highly distressing affect may be reminiscent of one’s feeling during abusive scenarios in childhood, automatically triggering dissociation.

The connection between dissociation and low mood is also noteworthy. A depressive state is often characterised by a critical internal dialogue, which might elicit dissociative responses- especially when dissociation occurred in response to critical caregivers. However, as this finding alluded to low mood and dissociation occurring in parallel, rather than indicating a potential direction, participants descriptions may capture periods where high levels of dissociation induced feelings of low self-efficacy and helplessness, contributing to low mood.

Distress was noted as a trigger, but also as a consequence of dissociation, which provoked a sense of helplessness and panic. Cognitive–behavioural models (Hunter et al., 2003) suggest that catastrophic misinterpretations exacerbate anxiety which in turn aggravates depersonalisation. Cognitive reappraisals are key in shaping emotional responses to dissociation (Hunter et al., 2003; Shipp et al., 2024), and the absence of normalising reappraisals, characteristic of adults who did not experience attachment figures as nurturing and containing, may elicit and maintain the sense of helplessness and lack of control described by participants in our sample.

In a minority of our sample, participants linked their experiences of detachment to psychotic-like experiences. Anomalous experiences are characteristic of clinical high-risk states and part of the psychosis prodrome for those who develop clinical psychosis (Davidsen, 2009; Nordgaard et al., 2017; Parnas, 2011), but at times are also reported in the context of dissociation in the absence of psychosis (Acunzo et al., 2020). Some participants identified experiencing tactile and visual hallucinations along depersonalisation (‘shutting down’, ‘disappearing’). For some participants, the sensations described were additionally thematically linked to experiences of trauma and were especially prominent among interviewees who had experienced sexual abuse. This link aligns with previous studies suggesting a causative role of dissociation in hallucinations (Černis et al., 2020) and a mediating effect in the relationship between DT and hallucinations (Bloomfield et al., 2021). Hallucinations can be regarded as dissociated experiences (Brewin et al., 2022; Moskowitz & Corstens, 2008) which result from a disruption of the integration of sensory and psychological experiences in the notion of the self (Longden et al., 2012), and may represent decontextualised memories of abuse among trauma survivors (Brewin et al., 2022). Although research has previously highlighted the strong association between dissociation and voices (Pilton et al., 2015) research on DT, dissociation and nonauditory hallucinations is limited. The non-auditory modality of hallucinations linked to dissociation in our sample supports the notion that the explanatory role of dissociation may extend to non-auditory hallucinations as identified in previous quantitative research (Longden et al., 2016; Nesbit et al., 2022; Wearne et al., 2022). Wearne et al. (2022) identified that a correlation between dissociation and visual hallucinations was found for individuals with PTSD and schizophrenia, but not for participants with PTSD or schizophrenia alone. Further research is necessary to elucidate whether the role of dissociation in psychosis differs across modalities, which experiences it interacts with, and to understand the neurobiological underpinnings of these experiences.

In addition to presenting a relationship with hallucinations, dissociation was also linked to experiences of suspiciousness, in keeping with a growing body of evidence that implicates dissociation in the experience of paranoia (Černis et al., 2020, 2021; Longden et al., 2020). Participants descriptions suggest a bridging role of anxiety in the link between their embodied experience of dissociation and mistrust towards others, adding to well established CBT models demonstrating a role for worry, panic and metacognitive beliefs on enhancing erroneous beliefs (Brett et al., 2009; Freeman et al., 2008; Wright et al., 2020). Although the majority of participants described dissociation as an antecedent of psychotic experiences, a few participants recognised dissociation as a way of coping with the distress caused from hallucinations, consistent with conceptualisations of dissociation as an escape in the context of threat and heightened affect (Foa & Hearst-Ikeda, 1996).

### 4.1. Strengths and limitations

The current study is the first to capture the lived experiences of the scantily studied dissociative phenomena in people at-risk of psychosis with a history of DT. Our exclusion of participants receiving psychiatric treatment minimises the potential confounding role of medication. A major strength of our methodology is the ability to provide insight into these heterogeneous and complex phenomena due to the rich dataset, serving as a valuable reference for clinicians. The study’s validity is strengthened by the large sample size for qualitative analysis and reliability is bolstered by using two coders and repeated discussions in theme development.

Nevertheless, a number of limitations need to be acknowledged. We relied on self-report measures to determine inclusion and exclusion criteria, such as retrospective reports of DT and reporting absence of a psychiatric diagnosis or psychiatric medication. Also, we did not account for previous histories of medication use or psychological treatment, or for unrecognised psychiatric diagnoses (e.g. undiagnosed dissociative disorder or post-traumatic stress disorder).

It is possible that inductive bias influenced our findings. For example, dissociative experiences on body ownership, depersonalisation, derealization, and dissociative amnesia were directly queried as part of the CAARMS, likely influencing the phenomenological descriptions of dissociation. However, several participants reported dissociative experiences outside of the specific questions. Another limitation concerns the potential selection bias in our sample. First, our recruitment strategy may have excluded individuals who are not active social media users. Second, our sample comprised of mostly young people, females, participants of White ethnicity and of higher education background. Although we aimed to exclude individuals receiving psychiatric intervention in order to target subclinical symptoms, some psychotic experiences arise along comorbid presentations, such as depression or anxiety. These characteristics may limit our finding generalisability to individuals with subclinical psychosis and DT across the UK. Yet, our sample’s young age may also reflect psychotic difficulties that have not progressed to a clinical level that requires psychiatric intervention.

Importantly, the cultural context and background of participants were also not explored, and our research’s conclusion apply to its Euro-American context. A frequent criticism is that the salience of or distress caused by dissociation largely depends on unique cultural and social norms, posing a challenge to universalist conclusions about the function of dissociation cross-culturally. Ethnographic research demonstrates great variability in conceptions of selfhood, the social acceptability of dissociative states/trance/possession (Bhavsar et al., 2016; Bourguignon, 1970), and embeddedness of trance and meditative states (Castillo, 2003) across cultural contexts.

### 4.2. Clinical implications and future directions

Our study has provided rich insights on putative mechanisms connecting DT, dissociation and psychosis, which would benefit from further quantitative exploration. The cross-sectional nature of our findings renders longitudinal and experimental research necessary, to clarify whether dissociative experiences contribute to the severity and distress of psychotic symptoms over time. It is essential to understand whether dissociation interacts with DT to predict psychotic symptom severity and distress, to improve the identification of patients who might necessitate additional intervention components to treat these experiences. Future research should explore the links between dissociation and affective experiences, such as panic, and emotion regulation processes, such as affect tolerance and interoception, among individuals with psychosis to inform psychological formulation and treatment.

Although our findings add to research increasingly highlighting the high frequency of dissociation in individuals with DT and psychosis, dissociative symptoms are rarely queried among patients with psychotic experiences. Together with the inherent phenomenological complexity and challenge to describe dissociation, its recognition by clinicians may be obstructed. Consequently, first, it is vital to screen and assess dissociative experiences in those reporting psychotic experiences with trauma histories. Second, our findings corroborate and extend previous proposals on the role of dissociation in maintaining psychotic and affective symptoms. Therefore, dissociation should be explored and incorporated in formulation and therapy for positive psychotic symptoms (Longden et al., 2020).

Increasing research and clinical recognition of the roles of trauma in psychosis has led to the development of different avenues for treatment, with multiple important preliminary trials currently underway. CBT for depersonalisation (Farrelly et al., 2024; Hunter et al., 2003), currently reported to be acceptable and feasible, offers a sensible avenue to address the links between dissociation and affective experiences, such as panic, low mood and affect regulation, but also meta-cognitive beliefs, which arose as predictors of distress among some participants. Another novel approach which has been co-produced with members of the Hearing Voices movement is the ‘Talking with Voices’ intervention (Longden et al., 2022), which encourages the dialogical engagement of voice hearers with their voices, conceptualising them through a dissociative framework (Longden et al., 2020; Moskowitz & Corstens, 2008).

As dissociation often arises along post-traumatic intrusions, trauma-focused therapies such as eye movement desensitisation and reprocessing or trauma-focused CBT, but also compassion focused therapy (Heriot-Maitland et al., 2019), could be a meaningful direction of treatment, with adaptations showing promising results for patients with psychosis (Burger et al., 2022; Peters et al., 2022; Varese et al., 2024). Finally, recent proposals suggest that mentalization, a process crucial for affect regulation and processing of information about the self and others, is central in shaping the influence of dissociation on psychotic experiences. Dissociation constitutes a key therapeutic target in preliminary studies of mentalization-based therapy for psychotic experiences (Bateman et al. 2023a; Weijers et al., 2020, 2021) and for trauma (Bateman et al., 2023b; Rüfenacht et al., 2023), which show promising results. As our findings highlight the clinically heterogeneous and complex phenomenological experience of dissociation, we believe that a tailored therapeutic approach that targets mechanisms, experiences and meanings unique to a persons’ formulation is essential. Although a range of approaches shows promise for the treatment of dissociation in those with psychosis and DT, future studies are necessary to establish the long-term effectiveness of these interventions using robust research designs.

## 5. Conclusion

Our analysis on interviews conducted with individuals with subclinical psychosis and histories of DT provided an in-depth exploration of the phenomenology of dissociative experiences, especially instances of depersonalisation, indicating that it is a frequent and highly distressing state. Interestingly, participants described a cascade of responses following trauma-related reminders or internal affective experiences characterised by intense emotions, helplessness and dissociation, with the embodied experience and meta-cognitive responses to dissociation acting as both a consequence and a trigger to intense distress. Further research is necessary to understand these mechanisms with larger quantitative and experimental and longitudinal designs. These findings underscore the importance of screening for dissociation among individuals with psychotic experiences and a history of trauma, and the collaborative exploration of these often-overlooked experiences for the development of clinical formulation and treatment.

## Supplementary Material

Supplementary_Material_1712.docx

## Data Availability

The data that support the findings of this study are not publicly available due to information their containing information that could compromise the privacy of research participants, but are available from the corresponding author [EM] upon reasonable request.
