# Supplementary material for: Dissociative experiences in individuals with subclinical psychosis and a history of developmental trauma: a qualitative study
Source: Eur J Psychotraumatol. 2025 Mar 11;16(1):2472473. doi: 10.1080/20008066.2025.2472473 (PMC11899206; doi:10.1080/20008066.2025.2472473)
Supplement: Supplementary_Material_1712.docx [file ZEPT_A_2472473_SM3821.docx]

**Supplementary Material 1**

Recruitment into the IMPACT study occurred through paid social media advertisements on Facebook, Twitter, and Instagram, where participants were invited to take part in an online study investigating the role of a) developmental trauma on adult mental health, and b) experiences of hearing voices, seeing things that others may not see, or feeling threatened by others.

Inclusion criteria were being over 18 years old, a UK resident, and fluent in English. As this constituted the community arm of the IMPACT study, we aimed to capture individuals with subclinical psychotic experiences. Therefore, participants currently receiving treatment from a mental health provider were excluded from the study.

Participants completed a battery of online self-report questionnaires, including information on sociodemographic variables and psychological tasks (reported elsewhere). As part of the self-report questionnaires, participants completed the Childhood Trauma Questionnaire^[28]^, the Community Assessment for Psychic Experiences (CAPE)-15^[29,30],^ and the International Trauma Questionnaire^[36]^.

The presence of a history of developmental trauma was operationally defined using the 28-item self-report Childhood Trauma Questionnaire (CTQ) ^[28],^ which assesses sexual abuse, physical abuse, emotional abuse, physical neglect, and emotional neglect. Scores on the CTQ range from 5 to 25 for each subscale and from 25 to 125 for the total score. We required participants to score "moderate" or higher in at least two trauma categories, or "severe" in at least one category, following the cut-off criteria established by Bernstein & Fink (1998). This was done to ensure specificity in identifying developmental trauma, given the known risk of over-reporting in online studies.

The Community Assessment of Psychic Experiences (CAPE)-15, a factorially robust measure with good psychometric properties and clinical utility in detecting individuals at Ultra High Risk of Psychosis ^[29, 30]^ , was used to screen participants for psychotic-like experiences. Each item on the CAPE-15 uses a 4-point Likert scale ranging from 0 (never) to 3 (nearly always). The total CAPE-15 score is divided by the total number of items (our online platform did not allow for missing responses) to produce a weighted score. To meet criteria for subclinical psychosis in the IMPACT study, participants had to achieve a weighted score of >1.47 on both the "frequency" and "distress" subscales of the CAPE-15.

Participants also completed the International Trauma Questionnaire to determine caseness for ICD-11 post-traumatic stress disorder (PTSD) and complex post-traumatic stress disorder (CPTSD). To meet the criteria for PTSD, participants had to endorse at least one or two items from each of the following subscales: re-experiencing, avoidance, sense of current threat, and functional impairment. To meet the criteria for CPTSD, participants not only had to meet the PTSD criteria, but also had to score at least one or two items in each of the following subscales: affective dysregulation, negative self-concept, disturbances in relationships, and impairment caused by these symptoms.

**Supplementary Material 2**

**1a. CAARMS questions related to dissociation**

***Depersonalization:***

*‘Do you ever experience yourself as being unreal, as if you were outside your own body? Or that part of your body did not belong to you?*’

***Derealization:***

*‘Have you ever had the feeling that things around you were unreal?’*

***Dissociative amnesia:***

*‘Have you ever found yourself somewhere without remembering how you got there?’*

**1b. CAARMS questions that could elicit responses relating to dissociation**

***Somatic Changes***

*‘Have you had the feeling that something odd is going on with your body that you can’t explain? What is it like? Do you feel that your body has changed in some way, or that there is a problem with your body shape?’*

*‘Do you ever get strange feelings in your body (e.g. feel that parts of your body have changed in some way, or that things are working differently)? Do you feel/think that there is a problem with some part, or all of your body, i.e. that it looks different to others, or is different in some way? How real does this seem?’*

***Visual Changes***

*‘Is there a change in the way things look to you? Do things somehow look different, or abnormal?’*

***Unusual Thought Content***

*‘Have you felt that something odd is going on that you can’t explain?’*

**Supplementary Material 3**

**Intrusions Interview**

The second part of the interview (Patel et al. 2007) focused on intrusive memories and images experienced in the past week. If more than 2 memories or images were identified, participants had to choose the 2 most distressing intrusions. Participants were additionally asked if they experienced intrusive thoughts or voices in the last week. Participants also provided ratings for the intensity and vividness of the intrusions, and the “nowness” of the intrusions (reported elsewhere).
